# Supplementary material for: The impact of sequencing depth on the inferred taxonomic composition and AMR gene content of metagenomic samples
Source: Environ Microbiome. 2019 Oct 24;14:7. doi: 10.1186/s40793-019-0347-1 (PMC8204541; doi:10.1186/s40793-019-0347-1)
Supplement: Supplementary file 1 — Additional file 1: Figure S1. Comparison of taxonomic classification output to simulated ground truth for CAMI gut metagenome dataset. (a) Taxonomic composition inferred using Kraken, Centrifuge, compared to ground truth, for ten simulated samples. The top 20 most abundant genera across all samples are shown in colour. (b) Relative species abundances compared to ground truth values for Kraken (blue) and Centrifuge (red). Lines show a linear best fit. [file 40793_2019_347_MOESM1_ESM.pdf]

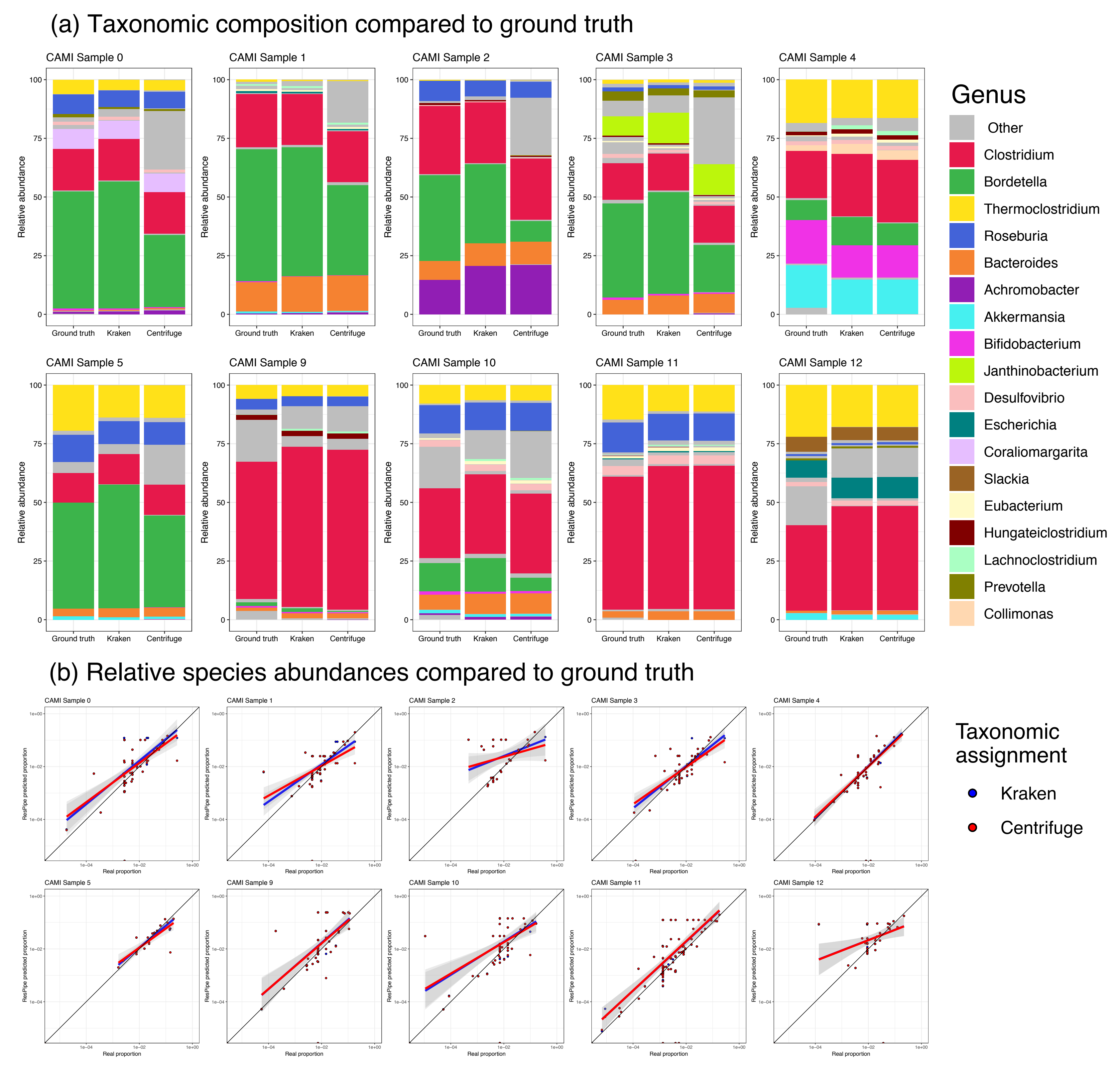

(a) Taxonomic composition compared to ground truth
CAMI Sample 0
CAMI Sample 1
CAMI Sample 2
CAMI Sample 3
CAMI Sample 4
CAMI Sample 5
CAMI Sample 9
CAMI Sample 10
CAMI Sample 11
CAMI Sample 12
Relative abundance
100
75
50
25
0
Ground truth
Kraken
Centrifuge
Genus
Other
Clostridium
Bordetella
Thermoclostridium
Roseburia
Bacteroides
Achromobacter
Akkermansia
Bifidobacterium
Janthinobacterium
Desulfovibrio
Escherichia
Coraliomargarita
Slackia
Eubacterium
Hungateiclostridium
Lachnoclostridium
Prevotella
Collimonas
(b) Relative species abundances compared to ground truth
CAMI Sample 0
CAMI Sample 1
CAMI Sample 2
CAMI Sample 3
CAMI Sample 4
CAMI Sample 5
CAMI Sample 9
CAMI Sample 10
CAMI Sample 11
CAMI Sample 12
ResPipe predicted proportion
Real proportion
1e+00
1e−02
1e−04
Taxonomic assignment
Kraken
Centrifuge
