# Supplementary figures and images for: The impact of sequencing depth on the inferred taxonomic composition and AMR gene content of metagenomic samples
Source: Environ Microbiome. 2019 Oct 24;14:7. doi: 10.1186/s40793-019-0347-1 (PMC8204541; doi:10.1186/s40793-019-0347-1)

(a) Kraken

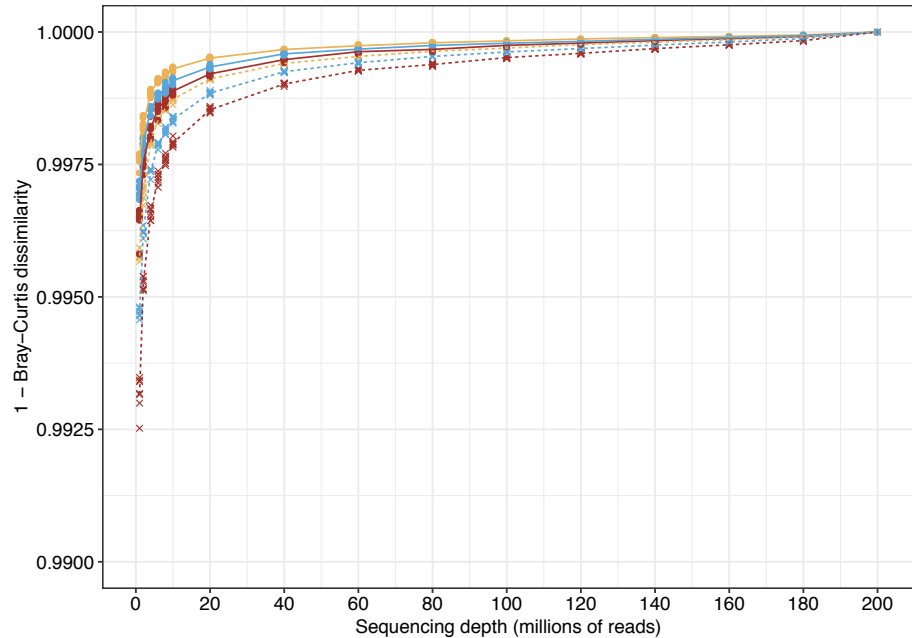

(b) Centrifuge

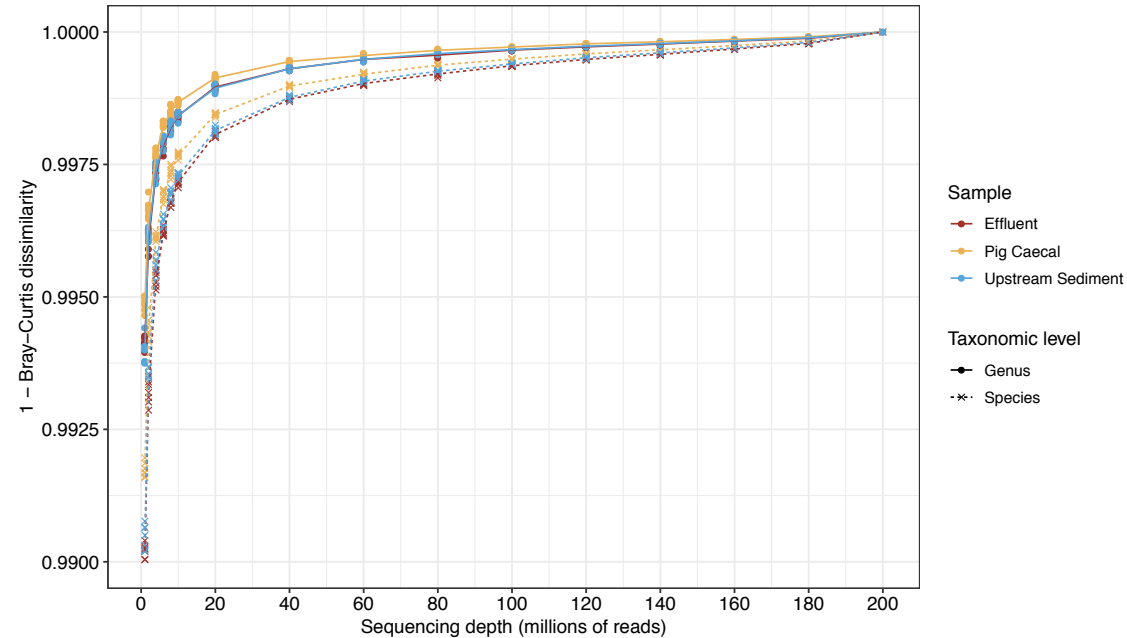

Supplement: Supplementary file 2 — Additional file 2: Figure S2. Effect of sequencing depth on Bray-Curtis dissimilarity to taxonomic composition of full sample. Results are shown for (a) Kraken and (b) Centrifuge for all samples at both genus and species level, comparing to the taxonomic composition at a depth of 200 million reads per sample. [file 40793_2019_347_MOESM2_ESM.pdf]
